# Molecular epidemiology and drug sensitivity of *Mycobacterium tuberculosis* in homeless individuals in the Addis Ababa city, Ethiopia

**DOI:** 10.1038/s41598-023-48407-8

**Published:** 2023-12-04

**Authors:** Tsegaye Shamebo, Balako Gumi, Aboma Zewude, Fikru Gashaw, Temesgen Mohammed, Muse Girma, Betselot Zerihun, Melak Getu, Sindew Mekasha, Muluwork Getahun, Biniam Wondale, Beyene Petros, Gobena Ameni

**Affiliations:** 1 Department of Microbial, Cellular and Molecular Biology, College of Natural and Computational Sciences, Addis Ababa University, PO. Box 1176, Addis Ababa, Ethiopia; 2 Aklilu Lemma Institute of Pathobiology, Addis Ababa University, Po. Box 1176, Addis Ababa, Ethiopia; 3 Ethiopian National Tuberculosis Reference Laboratory, Ethipian Public Health Institute, PO. Box 1242 or 5654, Addis Ababa, Ethiopia; 4 Department of Veterinary Medicine, College of Agriculture and Veterinary Medicine, United Arab Emirates University, PO Box 15551, Al Ain, United Arab Emirates; 5 Kotebe University of Education, Addis Ababa, Ethiopia; 6 Arba Minch University, Araba Minch, Ethiopia

**Keywords:** Microbiology, Molecular biology, Diseases, Risk factors

## Abstract

Although homeless segment of the society could be the hotspots for tuberculosis (TB) transmission, there is little data on TB in homeless individuals in Ethiopia. The objective of this study was to investigate the molecular epidemiology and drug sensitivity of Mycobacterium tuberculosis (M. tuberculosis) isolated from homeless individuals in Addis Ababa, Ethiopia. The study was conducted on 59 M. tuberculosis isolates, which were recovered by the clinical screening of 5600 homeless individuals and bacteriological examination of 641 individuals with symptoms of pulmonary tuberculosis (PTB). Region of difference-9 (RD9) based polymerase-chain reaction (PCR), Spoligotyping and 24-loci Mycobacterial Interspersed Repetitive Unit-Variable Number Tandem Repeat (MIRU-VNTR) typing were used for genotyping of the isolates. In addition, drug sensitivity test was performed on the isolates using BD Bactec Mycobacterial Growth Inhibition Tube (MGIT) 960. Fifty-eight of the 59 isolates were positive by spoligotyping and spoligotyping International type (SIT) 53, SIT 37, and SIT 149 were the dominant spoligotypes; each consisting of 19%, 15.5%, and10.3% of the isolates, respectively. The majority of the isolates (89.7%) were members of the Euro-American (EA) major lineage. MIRU-VNTR identified Ethiopia_3, Delhi/CAS, Ethiopia_2, TUR, X-type, Ethiopia_H37Rv-like strain, Haarlem and Latin-American Mediterranean (LAM) sub lineages. The proportion of clustering was 77.6% (45/58) in spoligotyping while it was 39.7% (23/58) in 24-loci MIRU-VNTR typing. Furthermore, the proportion of clustering was significantly lowered to 10.3% (6/58) when a combination of spoligotyping and 24-loci MIRU-VNTRplus was used. The recent transmission index (RTI) recorded by spoligotyping, 24-loci MIRU-VNTR typing, and a combination of the two genotyping methods were 58.6%, 27.6% and 5.2%, respectively. Young age and living in groups were significantly associated with strain clustering (P < 0.05). The drug sensitivity test (DST) result showed 8.9% (4/58) of the isolates were resistant to one or more first line ant-TB drugs; but multidrug resistant isolate was not detected. Clustering and RTI could suggest the transmission of TB in the homeless individuals, which could suggest a similar pattern of transmission between homeless individuals and the general population. Hence, the TB control program should consider homeless individuals during the implementation of TB control program.

## Introduction

Tuberculosis (TB), caused by members of the *Mycobacterium tuberculosis* complex (MTBC), is one of the leading causes of death from single infectious agent globally, accounting for about 1.5 million deaths and 10 million new cases each year^1,2^. An estimated 10.6 million people fell ill with TB in 2021, an increase of 4.5% from 2020 and 1.6 million people died from the disease^3^. The burden of drug-resistant TB also increased by 3% between 2020 and 2021, with 450,000 new cases of rifampicin-resistant TB (RR-TB) in 2021. The COVID-19 pandemic continues to have a damaging impact on access to TB diagnosis and treatment thereby leading to rise in the burden of TB disease. The progress made in the years up to 2019 has slowed, stalled or reversed and global TB targets are off track^3^.

Ethiopia is one of the 30 countries with a high burden of TB, MDR-TB and TB-HIV co-infection in the world^3^. In 2021, there were an estimated 119 incident TB cases per 100,000 populations in Ethiopia^3^. The current prevalence of MDR-TB in Ethiopia is 1.1% and 12% in new and previously treated TB cases, respectively^3^. On the other hand, a cross sectional study conducted on 228 individuals with chronic psychotic disorders at St. Amanuel Mental Specialized Hospital in Addis Ababa reported 9.8% drug resistant TB^4^.

Although homelessness is a worldwide crisis, the problem is more serious in developing countries like Ethiopia^5^. In Ethiopia, particularly in the major cities, homelessness is becoming a serious social problem^5,6^. The streets of Addis Ababa, the capital of Ethiopia, are said to be homes to a population of between 60,000 to100, 000 individuals^7,8^. Most of young homeless individuals residing in Addis Ababa city are migrants from rural areas in search of better opportunities although some have originated from the City itself^5^. Migration is predominantly caused by lack of support from social networks, lack of access to employment and income-generating opportunities, internal conflict, lack of access to affordable housing and lack of access to an effective social protection system^7,8^.

Homeless individuals live in conditions that are not conducive for living. They are exposed to risk factors of TB such as addiction to smoking, alcohol and drug^5,7^. In addition, the absence of shelter, HIV infection, overcrowding, lack of ventilation system and malnutrition increase the susceptibility of homeless individuals to TB infection. Understanding of local TB transmission would benefit both the infection control and management, which would ultimately result in a better patient care. Molecular epidemiological studies are very useful in identifying the TB bacilli strains that are circulating in specific geographic region or in specific group of human population such as the homeless individuals^9^. Furthermore, molecular epidemiological studies help in identifying the presence of ongoing active TB transmission as well as in differentiating reactivated infection from new infection. While investigation of drug sensitivity profiles of isolates would contribute to effective treatment of cases and alerts the TB control programs in containing MDR and extensive drug resistant isolates.

Studies from other countries show that strains of *M. tuberculosis* that infect homeless individuals are similar with those which infect the general community^9^. For instance, Haarlem, LAM and T sub-lineages were more frequently isolated from homeless individuals and general population in Colombia^9^. However, in Ethiopia although several molecular epidemiological studies conducted in the general population in different areas, no such study was conducted in marginalized homeless individuals. This study was initiated with the objective of investigating the molecular epidemiology of TB in homeless individuals who reside in the Addis Ababa city by using a combination of spoligotyping and 24-loci MIRU-VNTR genotyping methods^10^. The majority of the homeless individuals in Addis Ababa city were migrated to the City from different regional states of Ethiopia. Hence, they could be infected with different strains of *M. tuberculosis* which are circulating in different parts of the country. Therefore, the genetic diversity of the strains of *M. tuberculosis* that would be isolated from the homeless individuals in Addis Ababa could partly represent the diversity of *M. tuberculosis* strains at the national level. Moreover, evaluation of the drug sensitivity profiles of the *M. tuberculosis* isolated from the homeless individuals in Addis Ababa would generate valuable information on the magnitude of drug resistance, which would help the TB control program in planning effective control methods. Therefore, the objectives of this study were to investigate the molecular epidemiology and evaluate the drug sensitivity of *M. tuberculosis* isolated from homeless individuals in Addis Ababa city, Ethiopia.

## Materials and methods

### Study setting and design

A cross-sectional study was conducted among homeless individuals diagnosed with PTB between February 2019 and December 2020 in Addis Ababa, Ethiopia. All methods were performed in accordance with the relevant guidelines and regulations. During the study period Addis Ababa city Administration has established temporary shelters for homeless individuals. The shelters provided meal, bedding, clothing, health care service and counseling. Voluntary homeless individuals were enrolled from the streets of Addis Ababa city by the outreach program to get the shelter services. Eligible and voluntary homeless individuals were screened for the symptoms of PTB upon admission to the shelters. A total of 5600 homeless individuals were screened for the symptoms of PTB using WHO TB symptoms screening guidelines^11^. Xpert MTB/RIF assay and *M. tuberculosis* culture were performed on 641 sputum samples to isolate *M. tuberculosis*. *M.tuberculosis* isolates recovered from culture were examined with region of difference-9 (RD9)-based polymerase chain reaction (PCR), spoligotyping and 24-loci MIRU-VNTR typing. DST was performed using BD Bactec Mycobacterial Growth Inhibition Tube (MGIT) 960.

### Lowenstein-Johnson culture of sputum

Sputum samples were digested and decontaminated by the modified Petroff method^12^. Briefly, samples were decontaminated with equal volume of 4% NaOH for 15 min; the remaining volume was filled with 9 ml sterile phosphate buffer saline (PBS) and centrifuged at speed of 3000g for 15 min. A drop of phenol red was added to a pellet as pH indicator and neutralized using 10% HCl. Of the neutralized pellet, 0.25–0.5ml specimen was inoculated onto two slopes of Lowenstein-Johnson (LJ) media. One of the LJ media supplemented with 0.6% glycerol and the other with 0.75% pyruvate. Inoculated media were incubated at 37 °C for up to 8 weeks with weekly follow up. Culture was considered negative after 8 weeks if colony was not observed.

### DNA extraction

Extraction of mycobacterial DNA was performed by boiling a loop full of fresh grown bacterial colonies in 100 µL dH_2_O at 80 °C for 60 min. The extracted DNA samples were stored at – 80 °C until when spoligotyping and MIRU-VNTR typing were performed.

### Typing of *Mycobacterium tuberculosis* complex isolates

Three different molecular typing methods were used. First, region of difference 9 (RD9) based PCR was used for the differentiation of *M. tuberculosis* from the other members of MTBC. Secondly, the *M. tuberculosis* isolates were genotyped using spoligotyping for strain identification although it has low discriminatory power. Lastly 24-loci MIRU-VNTR was used for identification of the strains of *M. tuberculosis*. Thus, both spoligotyping and 24-loci MIRU-VNTR were used for identification of the strains of *M. tuberculosis*. A combination of spoligotyping and 24-loci MIRU-VNTR was used in order to get a higher resolution power than using either spoligotyping or MIRU-VNTR alone.

### Region of difference-9 based polymerase chain reaction

Region of difference (RD) 9-based PCR was performed on heat-killed cells to confirm the presence or absence of RD9 for species identification of *M. tuberculosis* from the other members of MTBC as previously described^13^. The PCR reaction was used three primers (RD9 flankF, RD9intR and RD9 flankR). Amplification of the mixtures was performed using a Thermal Cycler PCR machine. The PCR amplification product was run by electrophoresis in 1.5% agarose gel in 1 × Tris Borate-EDTA (TBE) running buffer at 110 V and 400 mA for 35 min. Ethidium bromide at a ratio of 1:10, 100 base pair (bp) DNA ladder and orange 6 × loading dye were used in gel electrophoresis and the gel was visualized. The results were interpreted as *M. tuberculosis* when a band size of 396 bp was observed (RD-9 positive), while detection of a band size of 575 bp was considered as positive for the other MTBC species. DNA from *M. bovis* BCG and *M. tuberculosis* H37Rv were used as positive controls, whereas distilled water was used as a negative control^13^.

### Spoligotyping

Spoligotyping was performed for MTBC isolates as described by Kamerbeek et al.^14^. The results of spoligotyping were interpreted in binary format and lineages were assigned using an updated version of the SIVITWEB (http://www.pasteur-guadeloupe.fr:8081/SITVIT2/) and the major lineages were analyzed using an online tool Run TB-Lineage http://tb.insight.cs.rpi.edu/run_tb_lineage.html. We also used a conformal Bayesian network (CBN) and knowledge based Bayesian network (KBBN) analyses to predict the major lineages and sub-lineages. Isolates which have similar pattern with those in the SITVIT database were assigned a SIT number while, new isolates were considered as “Orphan” strains^14^.

### Mycobacterial interspersed repetitive unit-variable number tandem repeat typing

The MTBC DNA samples were subjected to MIRU-VNTR typing as previously described^10^. Laboratory results of MIRU-VNTR typing were interpreted using the MIRU-VNTR*plus* database (http://www.miru-vntrplus.org) to determine MTBC lineages and relatedness. A minimum spamming tree (MST) was constructed. Previously identified Ethiopian strains were assigned manually to Ethiopia_2 and Ethiopia_3 based on the absence of spacer 13 and 10–19, respectively^15–17^. All the molecular typing tests were performed at the Aklilu Lemma Institute of Pathobiology (ALIPB), Addis Ababa University (AAU), Addis Ababa, Ethiopia.

### Drug susceptibility testing

Drug susceptibility test (DST) was performed at National Tuberculosis Reference Laboratory, Ethiopian Public Health Institute (EPHI), Addis Ababa; using liquid Mycobacterium Growth Indicator Tube system (MGIT) 960 as previously described^17^. The critical concentrations of the anti-TB drugs used for this study were: streptomycin (STM) 1µg, Isoniazid (INH) 0.1µg, Rifampicin (RIF) 1µg and Ethambutol (EMB) 5µg^18^.

### Statistical analysis

The data were analyzed using SPSS version 26 statistical software. The discriminatory power of genotyping methods were determined by the Hunter-Gaston Discrimination Index (HGDI)^19^.

$$ {\text{HGDI}} = 1 - \frac{1}{{N\left( {N - 1} \right)}}\sum\limits_{j = 1}^{s} {n_{j} \left( {n_{j} - 1} \right)} , $$

where N is the total number of isolates, s is the total number of different patterns and nj is the number of isolates belonging to the jth pattern. The allelic diversity (*h*) of each of the 24-loci MIRU-VNTR was determined from the MIRU-VNTR*plus*. Lineage identification of MTBC isolates was carried out by best match analysis and tree-based identification tools on the MIRU-VNTR*plus* database (http://www.miru-vntrplus.org)^20^. The classification of the isolates by MIRU-VNTR and spoligotyping was performed based on dendrogram provided in the MIRU-VNTR*plus* by the construction of a NJ based phylogenetic tree^20^. The genotype clustering rate was estimated by n-1 method^21^ according to the formula described earlier^22^ (n_c_-c)/n; where n is the total number of cases in the sample, c is the number of clusters and n_c_ is the total number of clustered cases in the cluster. The association between clustered strains and predictive variables were computed using logistic regression analysis. P-value < 0.05 was considered statistically significant.

### Ethical consideration

The protocol of the study was approved by the Addis Ababa University, College of Natural and Computational Sciences Institutional Review Board (IRB) (Ref. No. IRB/036/2018). Additionally, Ethical clearance was obtained from the Addis Ababa City Administration Health Bureau (Ref. No. A/A/H/3981/227). Then, a support letter was obtained from Addis Ababa City Administration, Labor and Social Affairs Bureau (Ref. No. A/A/L/S/66/116/163). Written informed consent was obtained from all the study participants who provided sputum samples after providing adequate information on the possible benefits and risks of the study. Patients found AFB or Xpert positive were referred to the clinics in the temporary shelters for treatment under the directly observable therapy short course (DOTS) program.

## Results

### Socio-demographic and clinical characteristics of the study participants

Out of total 5600 homeless individuals screened for PTB symptoms, 80.3% (4500/5600) were males. The median and mean ± SD age of study participants was 23 and 27.8 ± 9.5 years, respectively. Seventy percent (4869/5600) and 47.0% (2631/5600) of the study participants were not married and completed at least primary education, respectively. Over 90% (5060/5600) of the homeless individuals migrated to Addis Ababa City from the regional states of Ethiopia. Of the 5600 participants screened for PTB symptoms, 641 presumptive TB cases were identified on the basis of clinical signs and then further subjected to bacteriological examination (Table 1). Totally, 59 bacteriologically confirmed TB cases identified by the GeneXpert and LJ culture. Then, the 59 isolates were identified using RD 9-based PCR, spoligotyping and MIRU-VNTR typing.

**Table 1:** Socio-demographic characteristics of homeless individuals (n = 641) with PTB prevalence, Addis Ababa, Ethiopia, 2021.

Variable/characteristics		Total n (%)	TB culture positive n (%)	TB culture negative n (%)	P-value
Gender	Female	126 (19.7)	7 (5.6)	119 (94.4)	0.372
	Male	515 (80.3)	52 (10.1)	463 (89.9)	
Age	18–27	86 (13.4)	6 (7.0)	80 (93.0)	0.820
	28–37	314 (49.0)	34 (10.8)	280 (89.2)	
	38–47	176 (27.5)	15 (8.5)	161 (91.5)	
	48–57	42 (6.6)	3 (7.1)	39 (92.2)	
	58 and older	23 (3.7)	1 (4.3)	22 (95.7)	
Marital status	Single	557 (86.9)	49 (8.8)	508 (91.2)	0.652
	Married	39 (6.1)	6 (15.4)	33 (84.6)	
	Divorced	34 (5.3)	3 (8.8)	31 (91.2)	
	Widowed	11 (1.7)	1 (9.1)	10 (90.9)	
Educational status	Illiterate	255 (39.8)	30 (11.8)	225 (88.2)	0.332
	Primary school	301 (47.0)	22 (7.3)	279 (92.7)	
	Secondary school	71 (11.1)	6 (8.5)	65 (91.5)	
	Higher education	14 (2.2)	1 (7.1)	13 (92.9)	
Former residence	Urban	233 (36.3)	18 (7.7)	215 (92.3)	0.328
	Rural	408 (63.7)	41 (10.0)	367 (90.0)	
Medical history	New cases	56 (94.9)	56 (94.9)	0 (0.0)	
	Retreatment cases	3 (5.1)	3 (5.1)	0 (0.0)	

### Speciation of the isolates using RD 9-based PCR

Based on the RD9-based PCR, out of 59 bacteriologically confirmed isolates, 58 isolates were confirmed to be *M. tuberculosis.* One isolate did not give valid result, which could be due to insufficient DNA content or laboratory contamination. The isolates were further genotyped using spoligotyping and 24-loci MIRU-VNTR typing for the identification of lineages and strains.

### Spoligotyping based identification of lineages and sub-lineages

The results of spoligotyping are presented in Tables 2 and 3 while the raw data of the spoligotyping are presented in Supplementary 1. On the basis of spoligotyping, 81% (47/58) of the isolates belonged to 16 shared international types i.e. SIT numbers could be assigned to 81% of the isolates while SIT numbers could not be assigned to 19% (11/58) of the total isolates. As a result, these isolates were classified as orphans (Table 3). In terms of frequency, SIT53, SIT149 and SIT37 were the three most frequently identified spoligotypes, and consisting of 11, 9 and 6 isolates, respectively. Furthermore, TB-insight RUN TB-lineage analysis revealed that Euro-American (L4) (EA) was the most prevalent lineage consisting of 89.7% (52/58) of the total isolates. The second most prevalent lineage was East-African Indian (L3) consisting of 8.62% (5/58) isolates while Indo-Oceanic (L1) lineage was the least prevalent lineage as only one isolate belonged to it.

**Table 2:** Lineage of MTBC consisting of shared strains which were isolated from homeless individuals in Addis Ababa city.

SIT	Octal code	Binary code	Major lineages (TB insight)	Lineage (SITVITWEB)	Lineages (MIRU-VNR *plus*)	Isolates N (%)
25	703,777,740,003,171		EAI	CAS1_DELHI	Delhi/CAS	2 (3.4)
26	703,777,740,003,771		EAI	CAS1_DELHI	Delhi/CAS	1 (1.7)
37	777,737,777,760,771		EA	T3	Ethiopia_2	6 (10.3)
41	777,777,404,760,771		EA	Turkey	TUR	1 (1.7)
42	777,777,607,760,771		EA	LAM9	LAM	1 (1.7)
47	777,777,774,020,771		EA	H1	Haarlem	2 (3.4)
53	777,777,777,760,771		EA	T1	Ethiopia_3	11 (19.0)
54	777,777,777,763,771		EA	Manu2	TUR	5 (8.6)
118	777,767,777,760,771		EA	T1	Unidentified	2 (3.4)
119	777,776,777,760,771		EA	X1	X	2 (3.4)
121	777,777,775,720,771		EA	H3	Haarlem	1 (1.7)
149	777,000,377,760,771		EA	T3-ETH	Ethiopia_3	9 (15.5)
302	777,756,777,760,771		EA	X1	X	1 (1.7)
584	777,775,777,760,731		EA	T2	Unidentified	1 (1.7)
952	603,777,740,003,771		EAI	CAS1_DELHI	Delhi/CAS	1 (1.7)
1312	703,777,740,003,131		EAI	CAS1_DELHI	Delhi/CAS	1 (1.7)

*EA* Euro-American, *EAI* East-African Indian, *IO* Indo-Oceanic.

**Table 3:** Lineages consisting of orphan rains which were isolated from the homeless individuals in Addis Ababa city.

SIT	Octal code	Binary code	Major lineages (TB insight)	Lineage (SITVITWEB)	Lineage (MIRU-VNTR *plus*)	Isolates N (%)
Orphan-1	777,776,777,760,600		EA	Unknown	Haarlem	1 (1.7)
Orphan-2	777,777,377,770,771		EA	Unknown	Unidentified	1 (1.7)
Orphan-3	777,737,757,760,771		EA	Unknown	Ethiopian_H_37_RV-Like	2 (3.5)
Orphan-4	777,775,777,760,721		EA	Unknown	Ethiopian_H_37_RV-Like	2 (3.5)
Orphan-5	763,777,777,563,771		EA	Unknown	Delhi/CAS	1 (1.7)
Orphan-6	776,737,777,760,771		EA	Unknown	X	2 (3.5)
Orphan-7	777,777,774,030,771		IO	Unknown	Delhi/CAS	1 (1.7)
Orphan-8	777,777,775,730,771		EA	Unknown	Unidentified	1 (1.7)

*EA* Euro-American, *IO* Indo-Oceanic.

### Mycobacterial interspersed repetitive unit-variable number tandem repeat typing based identification of sub-lineages and allelic diversity

The raw data of MIRU-VNTR are presented in Supplementary 1 while representative images of MIRU-VNTR gel are presented in Supplementary 2A–D. The 24-loci MIRU-VNTR typing identified Ethiopia_3 (34.5%), Delhi/CAS (12.1%), Ethiopia_2 (10.3%), TUR (10.3%), X-type (8.6%), Ethiopia_H37Rv-like strain (6.9%), Haarlem (6.9%) and LAM (1.7%) sub-lineages. However, 8.6% of the isolates could not be assigned to lineages using MIRU-VNTR*plus* database. Based on the logistic regression analysis, young age (AOR = 4.8, 95% CI 1.56, 8.24) and living in a group were significantly (AOR = 7.2, 95% CI 4.64, 15.41) associated with the clustering of *M. tuberculosis* strains (Table 4).

**Table 4:**
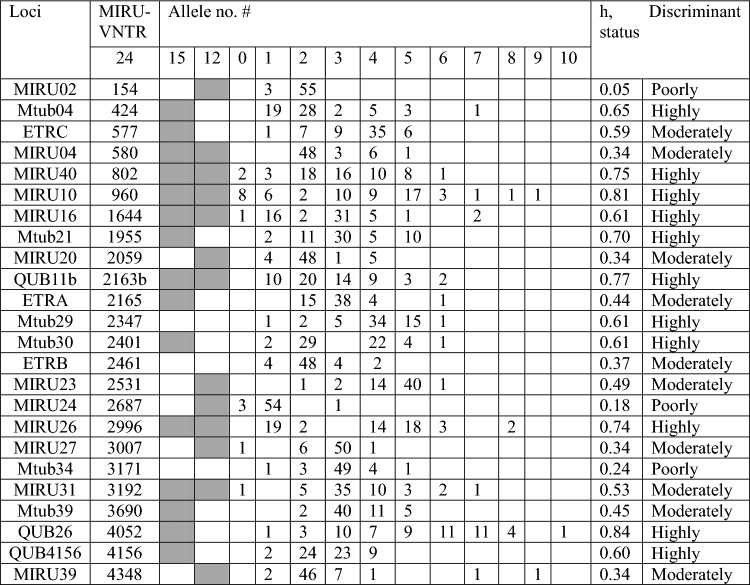
Occurrence of MIRU-VNTR alleles and allelic diversity of the MTBC strains isolated from homeless individuals in Addis Ababa.

Allelic diversity (*h*) for each MIRU-VNTR locus is presented in Table 5. MIRU-VNTR loci are classified based on their allelic diversity and ability to differentiate between the isolates. Eleven MIRU-VNTR loci (424, 802, 960, 1644, 1955, 2163b, 2347, 2401, 2996, 4052 and 4156) were highly discriminative (HGI > 0.6) while 10 MIRUVNTR loci (577, 580, 2059, 2165, 2461, 2531, 3007, 3192, 3690 and 4348) were moderately discriminative (0.3 < HGI < 0.6). The remaining three MIRU-VNTR loci (154, 2687 and 3171) were poorly discriminative (HGI < 0.3) (Table 5).

**Table 5:** Factors associated with the strains of MTBC isolated from homeless individuals in Addis Ababa.

Variable		Genotype		AOR (95% CI)	P-value
		Clustered, N (%)	Unique, N (%)		
Age	18–27	4 (57.1)	2 (28.6)	1	
	28–37	31 (91.2)	3 (8.8)	4.8 (1.56, 8.24)	0.001
	38–47	10 (66.7)	5 (33.3)	7.1 (0.96, 12.33)	0.068
	48–57	NA	3	NA	NA
Living with more than five individuals in one restricted place	No	5 (45.5)	6 (54.5)	1	
	Yes	40 (85.1)	7 (14.9)	7.2 (4.64, 15.41)	0.032
Past history of TB	No	42 (76.4)	13 (23.6)	1	
	Yes	2 (66.7)	1 (33.3)	1.84 (0.44, 6.23)	0.85
Isoniazid	Susceptible	33 (76.7)	10 (23.3)	1	
	Resistant	2	NA	NA	NA
Any resistance to FLDs	No	29 (70.7)	12 (29.3)	1	
	Yes	3 (75)	1 (25)	2.81 (0.92, 6.22)	0.74

### Minimum spanning tree analysis

**Figure 1:**
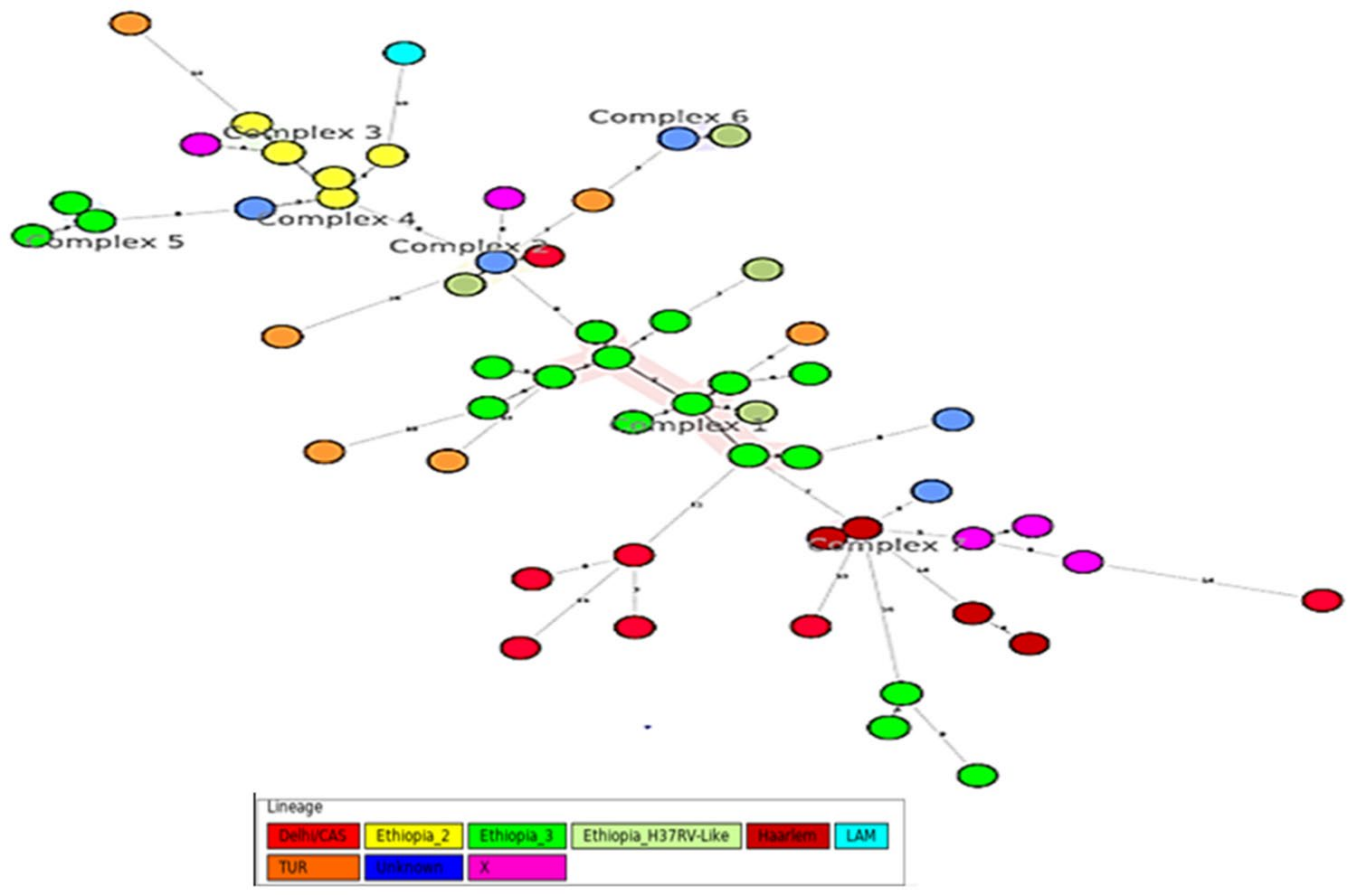
Minimum spanning tree based on 24-loci MIRU-VNTR profiles of 58 MTBC isolates from homeless individuals, Addis Ababa, Ethiopia. Sub-lineages are colored differently and individual pattern is represented by circle. The length of the branch represents the distance between patterns. A maximum locus difference within a clonal complex of MIRU-VNTR types is double locus variation. *M. canetti* was used as a reference strain from MIRU-VNTR *plus* database.

**Figure 2:**
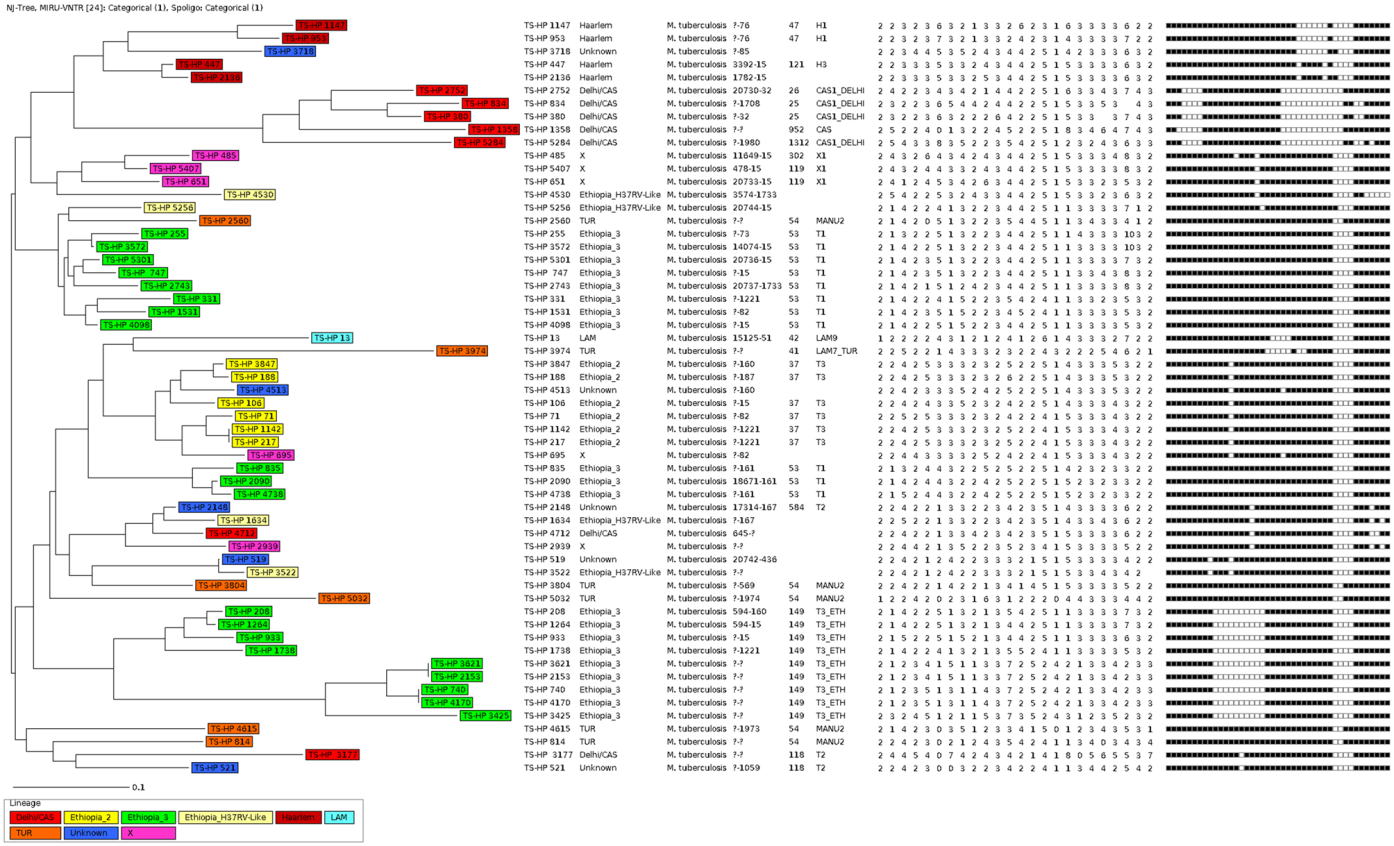
The phylogenetic tree of 58 MTBC isolates from homeless individuals, Addis Ababa, Ethiopia based on 24-loci MIRU-VNTR data. Neighbor-joining (NJ) tree showing the phylogenetic relationship of strains of MTBC isolated from Addis Ababa with the reference MTBC strains in the MIRU-VNTR*plus* database. The NJ tree was constructed using spoligotyping and 24-loci MIRU-VNTR data. MIRU-VNTR alleles and spoligo-patterns of 58 isolates were also represented along with the NJ tree. This phylogenetic tree was used to predict lineage of the new MTBC isolates from Addis Ababa. Web tools MIRU-VNTR*plus* (https://www.miru-vntrlus.org) was used to make phylogenetic tree.

Utilizing MIRU-VNTR data, Minimum spanning tree (MST) was constructed in which genotypes of isolates were linked based on double-locus variants (Fig. 1). Twenty-two genotypes (corresponding to 24 isolates) were grouped into 7 clonal complexes, leaving 34 singletons patterns. Clonal complexes 1 and 2 contained nine and three genotypes, respectively while clonal complexes 3, 4, 5, 6 and 7 each contained two genotypes, respectively. The MIRU-VNTR dendrogram constructed using http://WWW.miruvntrplus.org/MIRU/index is presented in Fig. 2. The dendrogram grouped the 58 isolates into 7 clonal groups. The 2nd group had a single isolate. The 1st, 3rd, 4th, 5th, 6th and 7th groups had 3, 9, 21, 10, 4 and 10 isolates, respectively.

### Discriminatory power of spoligotyping and 24-loci mycobacterial interspersed repetitive unit-variable number tandem repeat typing

The discriminatory power of spoligotyping and 24-loci MIRU-VNTR were 0.9864 and 0.9987, respectively. The analysis of spoligotyping resulted in 24 different spoligotype patterns of which 11 were clustered and 13 were unique. MIRU-VNTR*plus* analysis resulted in 55 different patterns of which 3 were clustered and 52 unique. The overall clustering rate based on a combination of spoligotyping and 24-loci MIRU-VNTR was 10.3% while the RTI was 5.2% based on a combination both genotyping methods (Table 6).

**Table 6:** Discriminatory power of spoligotyping, MIRU-VNTR typing and their combination.

Typing methods	Different patterns N (%)	Clusters (N)	Cluster size (N)	Clustered isolates N (%)	Unique isolates N (%)	Clustering rate (%)	RTI %	HGDI
Spoligotyping	24/58 (41.4)	11	2–16	45 (77.6)	13 (22.4)	77.6	58.6	0.9864
MIRU-VNTR-24	42/58 (72.4)	7	2–9	23 (39.7)	35 (60.3)	39.7	27.6	0.9987
Spoligotyping + MIRU-VNTR	55/58 (94.8)	3	2	6 (10.3)	52 (89.7)	10.3	5.2	0.9999

*HGDI* hunter gaston discrimination index.

### Drug susceptibility test

The drug susceptibility testing (DST) data was available for 76.3% (45/59) isolates. The result showed that 91.1% (41/45) of the strains were pan-susceptible while the remaining 8.9% (4/45) was mono-resistant to the first-line anti-TB drugs. Mono-resistance to either INH or SM was 4.4%. No isolate was MDR. All isolates that were resistant to first-line anti-TB drugs were tested for the second-line drugs and none were found resistant. Ethiopia_3 strains were exhibited higher percentage (6.7%) of resistance to at least one of the first-line anti-TB drugs as compared to the other strains.

## Discussion

This study was the first of its kind to investigate the molecular epidemiology and drug sensitivity of *M. tuberculosis* in the homeless individuals in Addis Ababa, Ethiopia. The study identified moderately diverse strains of *M. tuberculosis* in the homeless individuals. The clustering rate was also moderate in the homeless individuals. Clustering of strains was associated with young age and living in a group in one place. The isolates showed a moderate magnitude of drug resistance to first-line anti-TB drugs while no MDR-TB isolate was detected.

Based on the results of the spoligotyping, the Euro-American lineage (L4) (EA) was the most prevalent (89.7%) lineage identified in this study. This observation was consistent with the findings of previous studies, which showed the high prevalence of L4 in the world at large^23^ and also in Ethiopia^24–30^. Furthermore, two reviews on the genetic diversity of *M. tuberculosis* strains in Ethiopia indicated that the prevalence of L4 is higher than the prevalence of the other lineages^31,32^. Following to L4, the second most prevalently isolated lineage was L3. This observation is also consistent with the findings other studies reported from Ethiopia^24–30^. The reason for the high prevalence of L3 and L4 could be due to the fact that these lineages belong to the modern *M. tuberculosis* lineages which are characterized by high potential of transmissibility as compared to ancient *M. tuberculosis* lineages^33^. On top of L3 and L4, the Indo-oceanic lineage (L1) was isolated from the homeless individuals in Addis Ababa. Similarly, L1 was reported from Ethiopia earlier by other researchers^24^. However, the prevalence of L1 was very low in this study as compared to those of L3 and L4 although other researchers reported that L1 occurs abundantly in East Africa ^24^.

On the basis of MIRU-VNTR typing, T sub-lineages was the prevalent sub-lineage isolated by this study. This observation is consistent with results of previous studies in Ethiopia^16,34^. Earlier study documented that the T sub-lineages are specific to Ethiopia. And the result of this study thus re-affirmed the finding of the earlier study^15^. The T sub-lineages belong to L7 which has been isolated only from Ethiopia but not yet from other countries^15^. L7 is not widely spread even in Ethiopia and most commonly isolated from northeastern Ethiopia. Hence, because of its limited geographic scope in Ethiopia, the chance of its spread to other countries could be minimal. However, the present geographic scope of this lineage could change in the future thereby favoring its widespread in Ethiopia and neighboring countries. Ethiopia_3 was the predominant Ethiopia specific sub-lineage followed by the Ethiopia-2, similar to previous studies conducted in northwestern^16^ and eastern Ethiopia^35^. In contrast, studies conducted in southern Ethiopia^29^ reported that sub-lineages Ethiopia_2 the dominant sub-lineage in southern Ethiopia. Ethiopia_H37Rv-like genotype which shares a common ancestor with H37Rv laboratory reference strains was also isolated by the present study and earlier similar studies^36–39^.

Furthermore, Delhi/CAS was the second most prevalent sub-lineage detected in the homeless individuals in Addis Ababa. This result agrees with the results of several previous studies in Ethiopia^17,26,29,33,34,40^. Likewise, the Delhi/CAS sub-lineage was also prevalent in Tanzania^41^, Sudan^42^, Uganda^43^ and Kenya^44^. Although this lineage is presumed to be geographically specific to India and central Asia, the bi-directional socioeconomic relationship between Ethiopia and India might have increased the spread of Delhi/CAS lineage from India to Ethiopia. In this regard, Ethiopia was the first African country to open an embassy in New Delhi in 1948, which strengthened the relationship between the two countries as well as people to people interaction thereby favoring the transmission of the Delhi/CAS sub-lineage between Indians and Ethiopians. On top of this, isolation of the Delhi/CAS sub-lineage from homeless individuals in Addis Ababa could be substantiated by the theory of “Out of Africa”^45^ which stated that the origin of human beings and *M. tuberculosis* is East Africa. This theory thus indirectly suggested that Delhi/CAS and the other sub-lineages of *M. tuberculosis* could be found in East African countries like Ethiopia.

The proportion of Manu sub-lineage recorded by the present study was similar with those reported by previous studies conducted in Ethiopia^46^ and Egypt^47^. Although the Manu sub-lineage is less commonly reported from Ethiopia, its isolation could be associated with large number of Chinese in Ethiopia during the last three decades and their interaction with local people in different jobs such as construction and industrial enterprises. Similar to the result of the present study, the Turkey lineage was also isolated earlier in Ethiopia^26,29^ which could be due to the introduction of this lineage to Ethiopia from Turkey or Saudi Arabia to Ethiopia by movement of infected individuals in relation to growing economic partnership between Ethiopia and Turkish and/or Saudi Arabia. Furthermore, it could also be introduced to Ethiopia by the return of infected Ethiopian immigrants from Saudi Arabia/Turkey to Ethiopia^48^. Besides, the isolation of the Turkey sub-lineage in homeless individuals in Addis Ababa could be supported by the theory that stated East Africa is the origin MTBC members^45^.

The Haarlem sub-lineage is one of the common sub-lineages isolated in this study and it is believed to descend from the European and Middle East countries^44–51^. And its isolation from the Ethiopians is not surprising because of the long history of interactions between Ethiopians, and Europeans or people from the Middle East countries. Furthermore, the LAM sub-lineage is commonly isolated from Ethiopia as observed in this study^34,40^. Similarly, the LAM sub-lineage has been reported form the other east African countries such as Tanzania^44^, Uganda^52^ and Kenya^41^. Furthermore, similar to the results of the present study, the Haarlem, LAM and T sub-lineages were isolated from homeless individuals in Colombia^9^.

In the present study, majority of the MIRU-VNTR alleles were highly and moderately discriminant as evaluated by the allelic diversity (*h*) result, which in turn indicates the representativeness of the study population^10^. Furthermore, the finding of highly and moderately discriminant alleles suggest that the loci were suitable for genotyping of the isolates.

The results of MST and dendrogram analyses strains of *M. tuberculosis* isolated from homeless were consistent with the results of similar previous studies conducted in Ethiopia^17,53^.

Based on the generated data from spoligotyping and MIRU-VNTR, it is possible to say that these two genotyping methods can complement each other. But they have different precisions. For instance, SITVITWEB can only identify three Haarlem and five Delhi/CAS while MIRU-VNTR*plus* can identify four Haarlem and nine Delhi/CAS. These discrepancies probably associated with the algorithm used by such database. Nevertheless, the profiles of 19% (11/58) and 8.6% (5/58) of isolates could not match with the profiles strains that are found in SIVITWEB and MIRU-VNTR*plus* databases, respectively.

The proportion of clustering in this study by using spoligotyping and 24-loci MIRU-VNTR typing was in agreement with previous similar studies conducted in Ethiopia^16,48,54–56^. However, the clustering rate based on a combination of spoligotyping and 24-loci MIRU-VNTR typing was lower than other previous studies in Ethiopia^17,30^ and higher than a study reported from South Omo Zone Ethiopia^29^. The differences in clustering rate among studies could be due to variations in geography, population density, ethnicity and socio-economic diversity^57^. The significant clustering rate in our study could also be associated with presence of TB transmission among homeless individuals in Addis Ababa City which may due to overcrowding and other associated risk factors that favor the transmission of TB. Other previous studies have reported socio-demographic factors to be predictors of recent TB transmission, such as young age, ethnicity status, male sex, homelessness, incarceration, and overcrowding and drug abuse^57^. In our study, young age and overcrowding were significantly associated with clustering, indicating that homeless individuals are at risk of developing TB^9^, and also linked to recent transmission of TB in this population. Strains of the Ethiopia_3 sub-lineage were more likely to be clustered which could suggest a more frequent transmission of this strain in the homeless individuals.

Although the number of the isolates was small, 8.9% isolates was resistant to one or more first-line ant-TB drugs. Other studies in Ethiopia and in the other countries reported similar percentages of drug resistance^4,58–60^. Fortunately, MDR isolate was not detected in the isolates of this study. Similarly, other studies conducted earlier reported the absence of MDR isolates^57,58^. Nonetheless, the risk factor for the development of MDR in the homeless individuals is expected to be more likely.

### Limitations of the study

Only LJ was used for culturing *M. tuberculosis* and the small number of isolates were used for this study which could limit the precision of estimate of the relationship between homelessness and TB. Moreover, the lower sensitivity of phenotypic DST methods and lack of molecular confirmation for drug resistance strains of *M. tuberculosis* could also limit the precision of the results. However, regardless of its limitations, the result of the study could be considered as a valued input for the TB control program of the country.

## Conclusion

In the present study, the clustering rate of *M. tuberculosis* isolates was 10.3% based on a combination of spoligotyping and 24-loci MIRU-VNTR genotyping methods. Young age and living in groups were significantly associated with strain clustering. These observations could suggest that the majority of TB cases in the homeless individuals in Addis Ababa city are attributable to recent transmission. This could also suggest the potential role of homeless individuals in the transmission of TB to the general population. Besides, fortunately no MDR isolate was detected while a moderate magnitude of drug resistance to first-line anti-TB drugs was recorded. Based on this conclusive remarks, the Addis Ababa city TB control program is advised to consider homeless individuals in its TB control program.

### Supplementary Information

Supplementary Information 1.

Supplementary Information 2.

Supplementary Information 3.

Supplementary Information 4.

Supplementary Information 5.

Supplementary Legends.

## Data Availability

The datasets generated and/or analysed during this study are included in this published article in Supplementary 1 files.
