# Supplementary figures and images for: Molecular epidemiology and drug sensitivity of Mycobacterium tuberculosis in homeless individuals in the Addis Ababa city, Ethiopia
Source: Sci Rep. 2023 Dec 4;13:21370. doi: 10.1038/s41598-023-48407-8 (PMC10695943; doi:10.1038/s41598-023-48407-8)

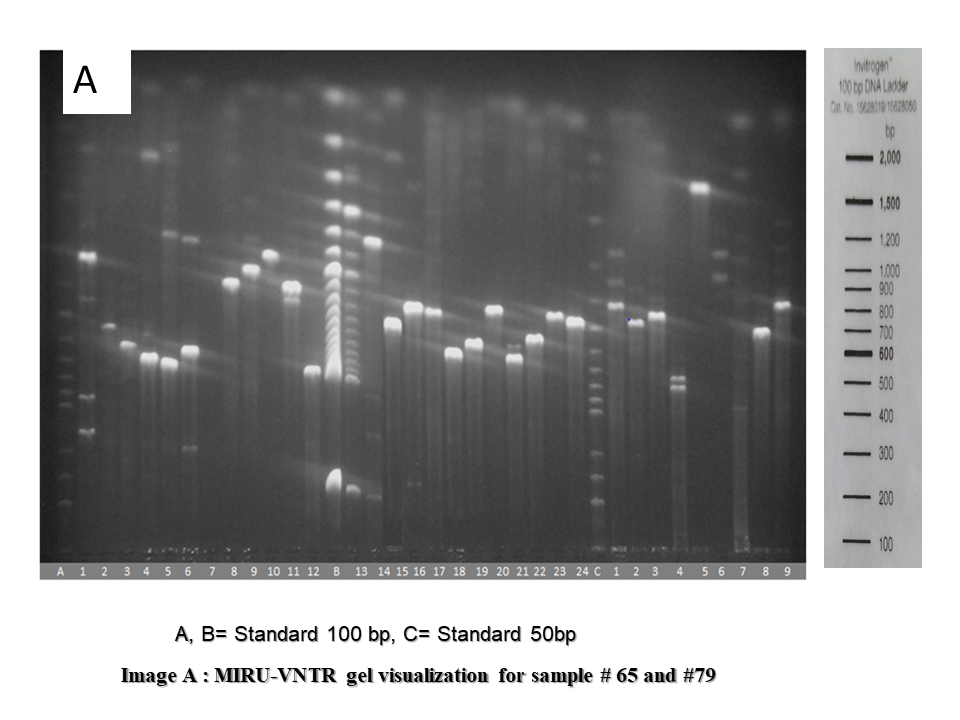

Supplement: Supplementary file 1 — Supplementary Information 1. [file 41598_2023_48407_MOESM1_ESM.tif]

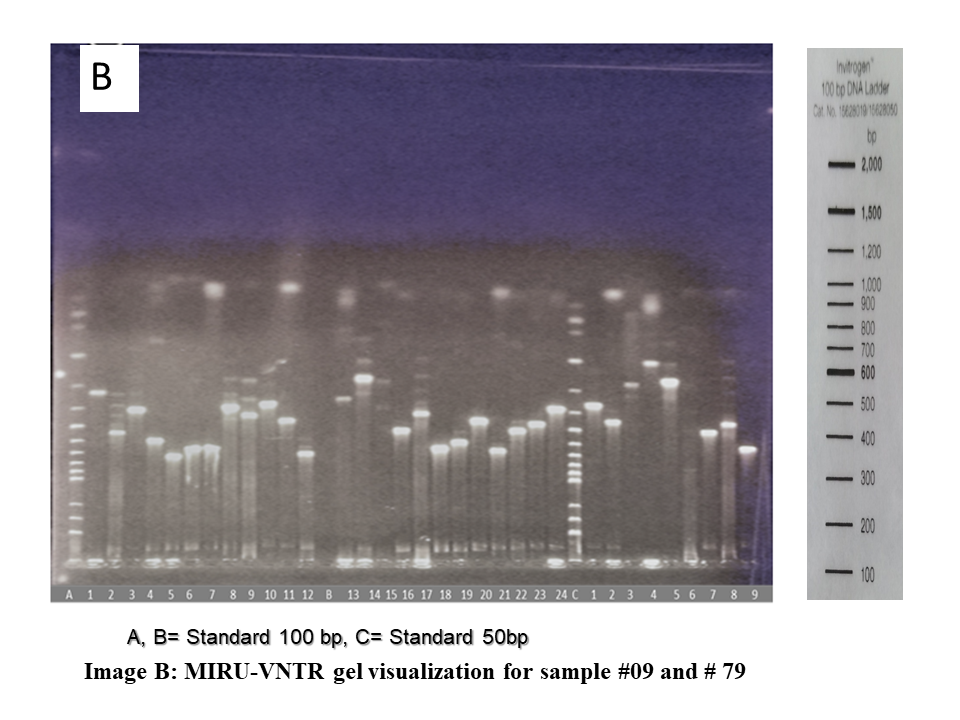

Supplement: Supplementary file 2 — Supplementary Information 2. [file 41598_2023_48407_MOESM2_ESM.tif]

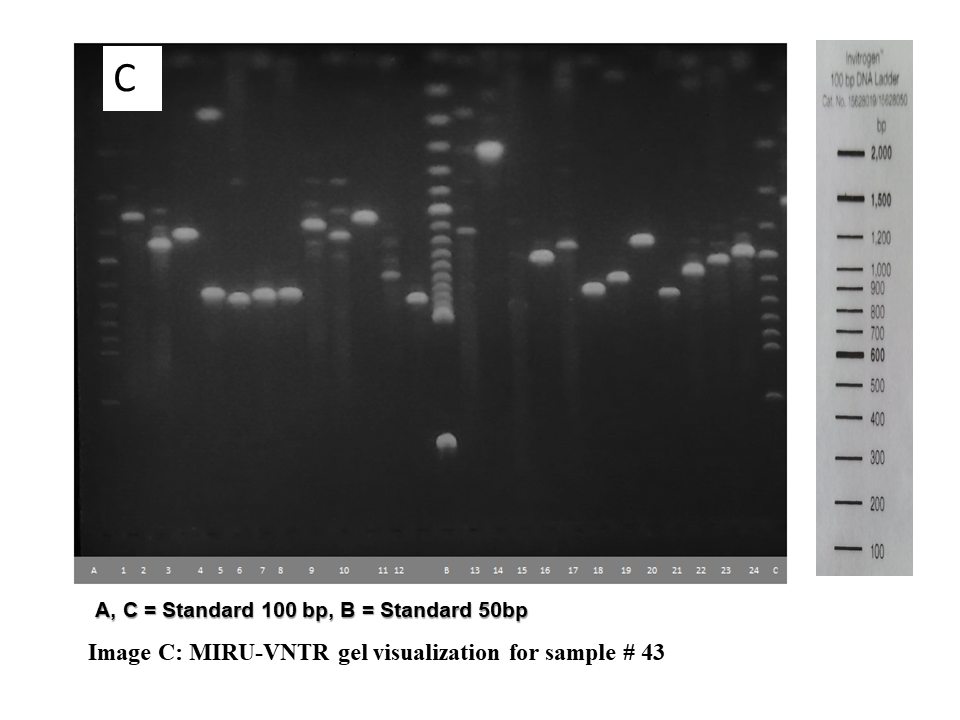

Supplement: Supplementary file 3 — Supplementary Information 3. [file 41598_2023_48407_MOESM3_ESM.tif]

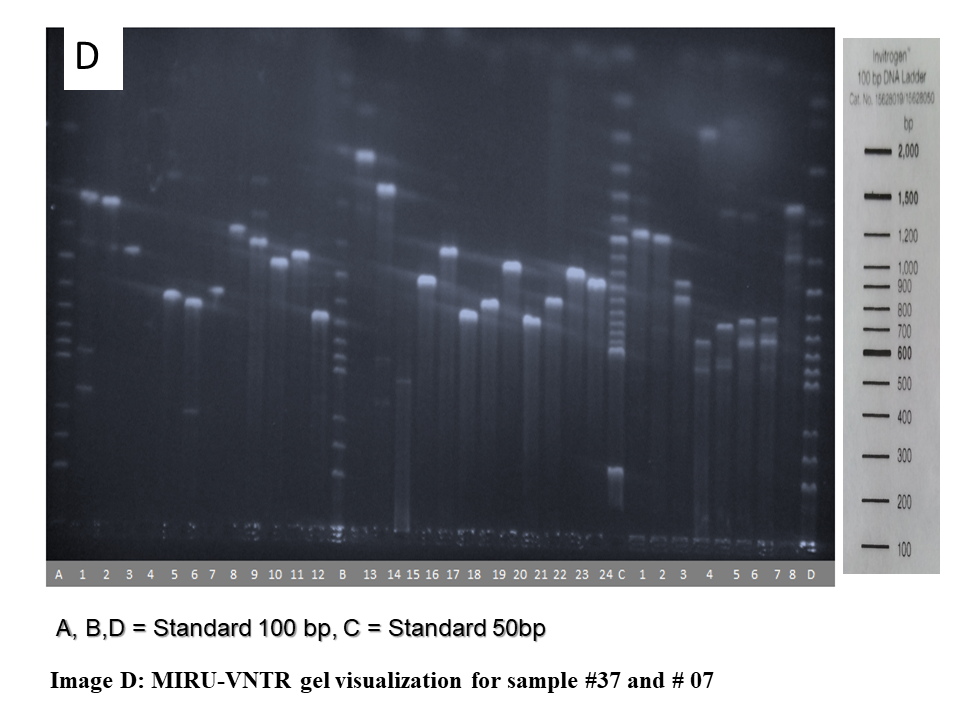

Supplement: Supplementary file 4 — Supplementary Information 4. [file 41598_2023_48407_MOESM4_ESM.tif]
