# Supplementary material for: Molecular epidemiology and drug sensitivity of Mycobacterium tuberculosis in homeless individuals in the Addis Ababa city, Ethiopia
Source: Sci Rep. 2023 Dec 4;13:21370. doi: 10.1038/s41598-023-48407-8 (PMC10695943; doi:10.1038/s41598-023-48407-8)
Supplement: Supplementary file 6 — Supplementary Legends. [file 41598_2023_48407_MOESM6_ESM.docx]

**Title of the supplementary files**

**Supplementary 1.** Raw data of MIRU-VNTR, spoligotyping and drug sensitivity tests of 58 M. tuberculosis isolated from homeless individuals in Addis Ababa city, Ethiopia

**Supplementary 2 (A-D).** Representative images of gel of 24-loci MIRU-VNTR of selected *M. tuberculosis* isolates of homeless individuals from Addis Ababa city, Ethiopia
